# The simultaneous administration of microplastics and cadmium alters rat testicular activity and changes the expression of PTMA, DAAM1 and PREP

**DOI:** 10.3389/fcell.2023.1145702

**Published:** 2023-03-09

**Authors:** Massimo Venditti, Majida Ben Hadj Hassine, Imed Messaoudi, Sergio Minucci

**Affiliations:** ^1^ Dipartimento di Medicina Sperimentale, Sez. Fisiologia Umana e Funzioni Biologiche Integrate “F. Bottazzi”, Università degli Studi della Campania “Luigi Vanvitelli”, Naples, Italy; ^2^ Laboratoire LR11ES41 Génétique Biodiversité et Valorisation des Bio-ressourcés, Institut Supérieur de Biotechnologie de Monastir, Université de Monastir, Monastir, Tunisia

**Keywords:** microplastic, cadmium, endocrine disrupters, prothymosin alpha, cytoskeleton, DAAM1, PREP, testis

## Abstract

This paper confirms the damaging effects produced by MP and Cd on testicular activity in the rat. Oral treatment with both chemicals resulted in testicular damage, documented by biomolecular and histological alterations, particularly by impaired morphometric parameters, increased apoptosis, reduced testosterone synthesis, and downregulation of the steroidogenic enzyme 3β-HSD. We also demonstrated, for the first time, that both MP and Cd can affect the protein level of PTMA, a small peptide that regulates germ cell proliferation and differentiation. Interestingly, the cytoarchitecture of testicular cells was also altered by the treatments, as evidenced by the impaired expression and localization of DAAM1 and PREP, two proteins involved in actin- and microtubule-associated processes, respectively, during germ cells differentiation into spermatozoa, impairing normal spermatogenesis. Finally, we showed that the effect of simultaneous treatment with MP and Cd were more severe than those produced by MP alone and less harmful than those of Cd alone. This could be due to the different ways of exposure of the two substances to rats (in drinking water for Cd and in oral gavage for MP), since being the first contact in the animals’ gastrointestinal tract, MP can adsorb Cd, reducing its bioavailability through the Trojan-horse effect.

## 1 Introduction

Reproductive activity is one of the main attributes of living beings, as it is fundamental to species survival and genetic variability. To successfully achieve it, the production of good quality gametes, capable of fertilizing or being fertilized, is of primary importance (Serrano et al., 2022). Male gamete differentiation occurs during spermatogenesis, a genetic and molecular program during which immature GC undergo several stages of cell division (mitotic and meiotic) and then, by intimate biochemical and morphological modification, to produce mature SPZ (Dunleavy et al., 2019). The entire progression is regulated not only by a sophisticated network of autocrine/paracrine/endocrine factors, but also by modulating the expression of several exclusive testicular genes (Venditti et al., 2020a; Miyaso et al., 2022).

With the aim of expanding knowledge about the mechanisms of spermatogenesis and human reproduction, we have previously characterized some proteins, such as PTMA (Venditti and Minucci, 2017; Minucci and Venditti, 2022; Venditti et al., 2022), and two cytoskeleton-related proteins, DAAM1 (Minucci and Venditti, 2022; Pariante et al., 2016a; Venditti et al., 2020b; c; Venditti et al., 2021a) and PREP (Santillo et al., 2019; Venditti and Minucci, 2019; Venditti et al., 2020b; Venditti et al., 2021a; Minucci and Venditti, 2022), in the testis, suggesting their involvement in the proliferative and differentiative stages of gametogenesis. In particular, PTMA, one of the most acidic mammalian polypeptides (3.5 isoelectric point), is an anti-apoptotic factor associated with post-meiotic GC progression and SPZ interaction with the oocyte (Venditti and Minucci, 2017). DAAM1 is a formin that promotes the nucleation of unbranched actin filaments (Pariante et al., 2016a; Venditti et al., 2018; Venditti et al., 2020b; Venditti et al., 2020c; Venditti et al., 2021a; Minucci and Venditti, 2022), while PREP is a serine protease associated with microtubules (Santillo et al., 2019; Venditti et al., 2019; Venditti and Minucci, 2019; Venditti et al., 2020b; Venditti et al., 2021a; Minucci and Venditti, 2022).

It is well known that since spermatogenesis is a very complex mechanism, even the slightest “mistake” can cause problems in its proper functioning, resulting in reduced sperm quality, and thus infertility (Vander Borght and Wyns, 2018). Indeed, many studies have described a progressive decline in fertility rates over the past 50 years, ranging from the 7%–8% observed in the early 1960s to the 15% we are witnessing today (Levine et al., 2017; Levine et al., 2022). Although in many cases, the causes of male infertility can be attributed to well-known factors (genetic abnormalities, lifestyle), for most cases the etiology is still unknown (idiopathic infertility) (Serrano et al., 2022). It should be considered that, among the factors affecting sperm quality, exposure to environmental pollutants is undoubtedly one of the main causes since, although male infertility is a worldwide health problem, its incidence is higher in more technologically advanced and developed countries (Marić et al., 2021; Selvaraju et al., 2021).

Among the excess of hazardous substances released into the environment, MP can affect human, animal, and plant health. These particles result from the degradation of plastic objects into fragments less 5 mm in size, which can be widely distributed throughout environment compartments. MP can be easily ingested by a variety of organisms, especially in the aquatic environment, entering the food web, therefore, the non-occupational population is inevitably, and often unawares, continuously exposed to MP (EFSA, 2016; Lehner et al., 2019). Scientific knowledge about the health risks posed by MP pollution on wildlife and humans is growing but, at the same time, remains extremely limited, specially when considering critical biological functions, such as reproduction. MP have recently been shown to affect mammalian testicular physiology by inducing inflammation, oxidative stress, impairment of the SE cytoarchitecture and BTB integrity, ultimately, leading to abnormal differentiation of mature gametes (Hou et al., 2021; Jin et al., 2021; 2022; Li et al., 2021; Wei et al., 2021; Ilechukwu et al., 2022; Wen et al., 2022).

Another aspect to be considered is that MP inevitably coexist with other chemicals in the environment and, due to their high surface area/volume ratio and hydrophobicity, can adsorb and transport them, thanks to a phenomenon called the “Trojan horse” effect; this influences their behavior once ingested, altering the uptake of chemicals and their distribution in tissues, with synergistic and/or antagonistic effects (He et al., 2021).

This paper focuses on the effects of MP and/or Cd on several parameters related to rat testicular activity, such as steroidogenesis, apoptosis, and, for a deeper comprehension of their effects, the analysis was also extended to the expression and localization of PTMA, DAAM1 and PREP. Indeed, many works reporting the effects of environmental pollutants on reproductive physiology have shown that one of the main targets of these substances is exactly the cytoskeleton of testicular cells (Wang et al., 2020a; Wang et al., 2020b; Belgacem et al., 2022), and we recently demonstrated that DAAM1 and PREP are specific targets of Cd toxicity (Chemek et al., 2018; Venditti et al., 2020d; 2021b), however; to our knowledge, no paper has studied the impact of MP, alone or given in combination with Cd, on DAAM1 and PREP. Regarding PTMA, we recently showed its significant role, as anti-apoptotic factor, in regulating cell cycle progression occurring during normal and/or pathological cell differentiation of mammalian testis (Venditti et al., 2022); but, to date, no evidence demonstrated whether PTMA may be a target of environmental pollutants, as MP and/or Cd.

Finally, this is because much evidence emphasizes the importance of using *in vivo* and *in vitro* models to predict reproductive fitness in humans exposed to environmental pollutants, as well as to better comprehend the mechanisms that regulate cellular and molecular events occurring during spermatogenesis (Li et al., 2016; Gao et al., 2021; Wang et al., 2022a; Wang et al., 2022b).

## 2 Materials and methods

### 2.1 Animals, experimental design, and sample collection

Thirty-two-two-months-old male Wistar rats, weighting 222 ± 18.97 g, were housed individually in stainless steel cages under controlled conditions of temperature (22° ± 2°C), light (hours light/dark schedule), and humidity (55% ± 20%). The animals had free access to food and water *ad libitum*. The rats, randomly divided into four groups (*n* = 8 each), were treated as follows: 1) control, which received daily oral gavage with ultrapure water; 2) 0.1 mg pristine polystyrene MP-treated (#PS/Q-R-KM491; GmbH, Berlin, Germany); 3) Cd + MP-treated (50 mg CdCl_2_/L + 0.1 mg MP); 4) Cd-treated (50 mg CdCl_2_/L in drinking water; Sigma-Aldrich, Milan, Italy). MP solution was prepared by diluting l mg of MP in 5 mL of MiliporeMili-Q water and processed by ultrasonic vibration, 0.5 mL of the resulting solution was administered by oral gavage once daily (0.1 mg/day corresponding to 1.5 × 10^6^ particles/day). The concentration of MP was chosen in accordance with Deng et al. (2017); Haddadi et al. (2022), while that used for Cd was the same as previously utilized in our studies (Chemek et al., 2018; Venditti et al., 2020d; Venditti et al., 2021b; Venditti et al., 2021c). The two separate exposure modes (drinking water for Cd and oral gavage for MP) were chosen to avoid early adsorption of Cd by MP.

Animals were treated for 30 days and weighted every 5 days. At 31st day, blood was obtained by cardiac puncture in heparinized tubes, then the animals were euthanized with 4% chloral hydrate (i.p. 10 ml/kg). Blood was centrifuged at 3,500 rpm for 15 min at 4°C, and plasma was collected and stored at −80°C for T assay. From each rat, the left testis was immersed in 10% formol buffer for histological studies, and the right testis was stored at −80°C for biomolecular studies. The rats were housed in accordance with the EEC 609/86 Directives regulating the welfare of experimental animals. The experimental protocol was approved by the ethics committee of Institute of Biotechnology, University of Monastir, (Ref: CER-SVS/ISBM022/2020).

### 2.2 Serum T concentration

Serum T level was quantified using the ELISA kit for T (#DE1559; Demeditec Diagnostics GmbH). The detection limit for T was defined at 0.083 ng/ml, and the optical density was read at 450 nm. An ELISA reader (#RT-2100C; Rayto Life and Analytical Sciences Co., Shenzhen, China) automatically calculated the concentration.

### 2.3 Histology and TUNEL assay

Fixed testes were dehydrated in increasing concentrations of ethanol before paraffin embedding. Serial sections five-μm thick were stained with hematoxylin/eosin. For histopathological evaluation, 30 seminiferous tubules/animal, for a total of 240 tubules per group, were counted under light microscope (Leica DM 2500, Leica Microsystems, Wetzlar, Germany). Photographs were taken using the Leica DFC320 R^2^ digital Camera (Leica Microsystems, Wetzlar, Germany).

Apoptotic cells were investigated by TUNEL-assay using the DeadEnd™ Fluorometric TUNEL System (#G3250; Promega Corp., Madison, WI, United States) following manufacturer’s protocol. The sections were then counterstained with mounting medium with DAPI (#ab104139; Abcam, Cambridge, United Kingdom) to mark the cell nuclei. The sections were observed and acquired with the optical microscope (Leica DM 5000 B + CTR 5000, Leica Microsystems, Wetzlar, Germany) with UV lamp and saved with IM 1000 software. For the count of the percentage of TUNEL positive cells, 30 fields/samples were considered and analyzed, for a total of 240 per group.

### 2.4 RT-PCR analysis

Total RNA was extracted from testicular samples using RNA-Xpress Reagent (#MB601; HiMedia Laboratories GmbH; Einhausen, Germany) and processed as described in Venditti et al. (2021b). For details on the used primers, see Supplementary Table S1. The relative amount of the Daam1 and Prep mRNAs were calculated from the values of Daam1/Act and Prep/Act ratio values and represented as OD units. All the RT-PCR were performed in triplicate.

### 2.5 Protein extraction and WB analysis

Proteins were extracted from the testis according to Ergoli et al. (2020). Lowry assay was used to measure protein concentration (Lowry et al., 1951). For each lane, 40 μg of proteins were separated SDS–PAGE by at nine or 15% and transferred to Hybond-P PVDF membranes (#GE10600023; Amersham Pharmacia Biotech, Buckinghamshire, United Kingdom). For details on the used antibodyes, see Supplementary Table S2. All signals, including those used to obtain the Bax/Bcl-2 ratio, were quantified by densitometric analysis using ImageJ software (version 1.53 g) and adjusted with respect to *ß*-Actin levels. All the WBs were performed in triplicate.

### 2.6 IF analysis

For IF staining, testis sections were processed according to Venditti and Minucci (2022). For details on the used antibodies, see Supplementary Table S2. The cells’ nuclei were marked with mounting medium with DAPI. The sections were observed and acquired under the optical microscope (LeicaDM5000 B+ CTR 5000) with a UV lamp and saved with IM 1000 software (version 4.7.0). Densitometric analysis of the IF signal was performed with ImageJ doftware and 30 fields/samples, for a total of 240 per group, were considered.

### 2.7 Statistical analysis

Data are reported as mean ± SEM. Differences between the groups were considered statistically significant at *p* < 0.05. Analyses were performed using one-way ANOVA; Tukey’s *post hoc t*-test was applied when appropriate with Prism 5.0, GraphPad Software (San Diego, CA, United States).

## 3 Results

### 3.1 Histological study

**FIGURE 1:**
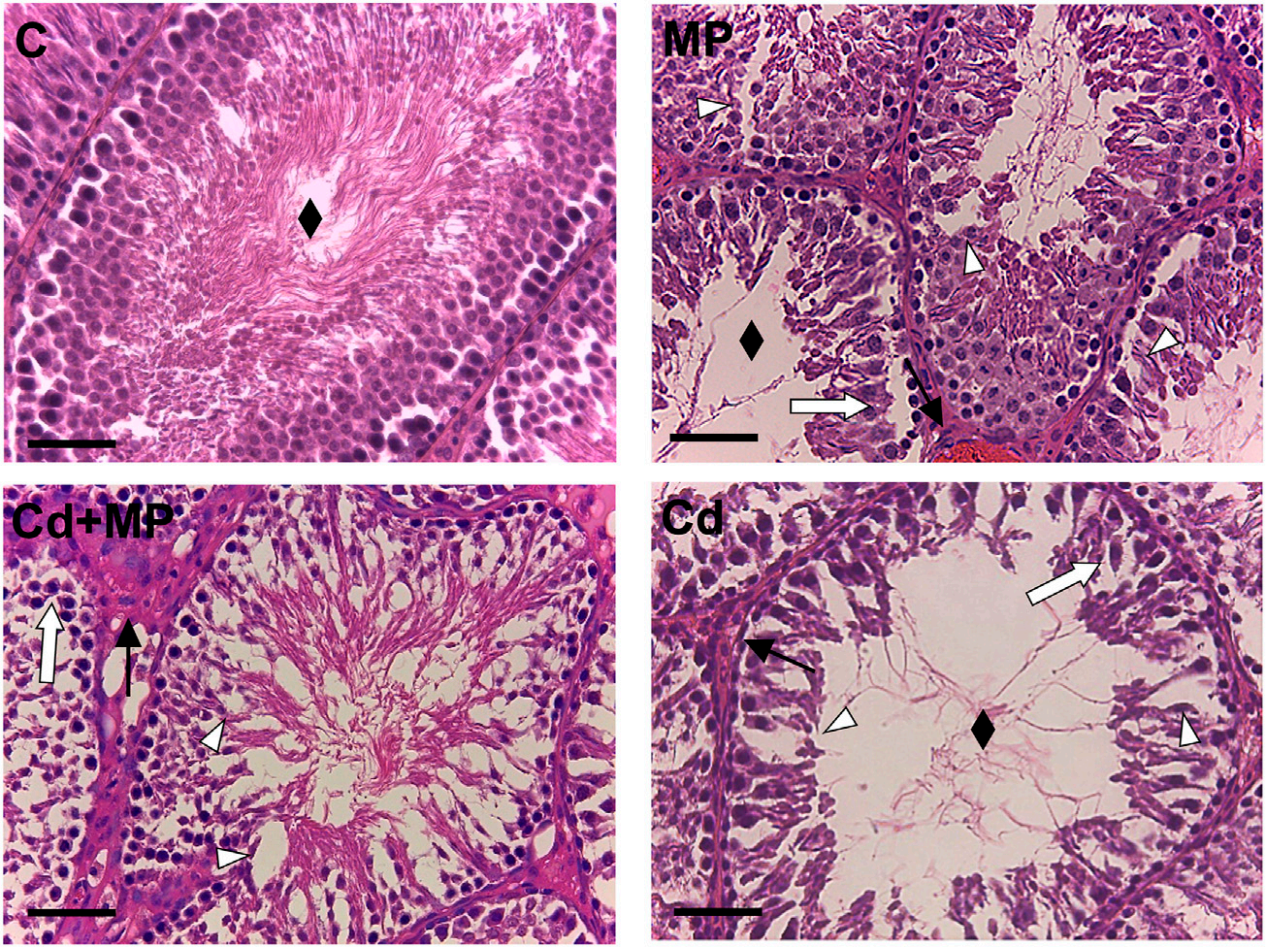
Hematoxylin-eosin staining of control, MP and/or Cd-treated rat testis. Rhombus: tubules lumen; White arrow: GC desquamation; Triangle: space between GC; Black arrow: dilatation of blood vessels. Scale bars represent 40 µm.

Representative images of rat testis sections are shown Figure 1. The control testis exhibited a regular SE, with GC in all different stages of differentiation and the tubular lumens filled with mature SPZ (rhombus), as well as a normal interstitial compartment, with the presence of regular LC and blood vessels. Abnormal seminiferous tubules, with general disorganization of the epithelium, were evident in all treated groups, as indicated by loss of contact and the presence of abundant empty spaces between cells (white triangle) other than desquamation of GC from the basement membrane (white arrow) and congestion (black arrow).

The histology results were confirmed by the analysis of morphometric parameters (Table 1), which showed that tubule diameter, epithelium thickness, and the percentage of tubular lumens occupied by SPZ were significantly lower in the MP (*p* < 0.05), Cd + MP (*p* < 0.01) and Cd (*p* < 0.001) groups as compared to the control.

**TABLE 1:** Effect MP and/or Cd on testicular morphometric parameters. Values are expressed as mean ± SEM from six animals in each group. a vs. b: *p* < 0.05; a vs. c: *p* < 0.01; a vs. d: *p* < 0.001; b vs. c: *p* < 0.05; b vs. d: *p* < 0.01; c vs. d: *p* < 0.01.

**Groups**	**C**	**MP**	**Cd + MP**	**Cd**
Tubules Diameter (µm)	127.09 ± 9,93^a^	92.99 ± 5,26^b^	80.87 ± 4,32^c^	62.92 ± 2,63^d^
Epithelium Thickness (µm)	45.47 ± 1,91^a^	33.29 ± 2,49^b^	28.38 ± 0,28^c^	22.68 ± 1,09^d^
Empty Lumen (%)	31.4 ± 3,6^a^	44.9 ± 2,1^b^	50.3 ± 1,9^c^	59.3 ± 2,1^d^

Interestingly, the results showed that in the Cd-treated group, all the above parameters had the lowest values (Table 1).

### 3.2 Effect of MP and/or Cd testicular steroidogenesis

**FIGURE 2:**
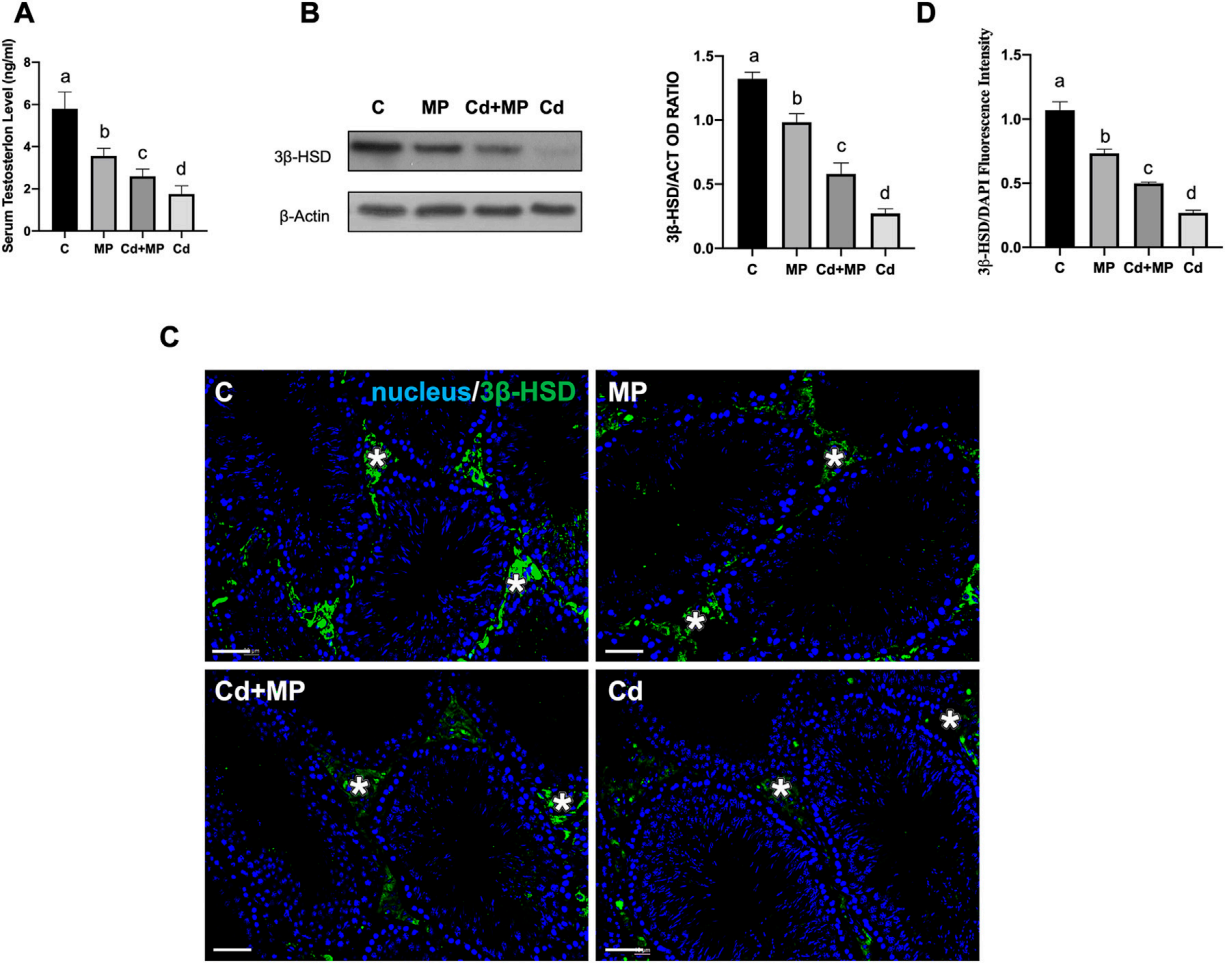
Steroidogenesis analysis of control, MP and/or Cd-treated rat testis. **(A)** Serum T level in animals treated with MP and/or Cd. **(B)** WB analysis showing the expression of 3β-HSD (42 kDa) in the testes of animals treated with MP and/or Cd. Histogram shows its relative protein levels. Data were normalized with *ß*-Actin and reported as OD ratio. **(C)** IF analysis of 3β-HSD (green) in testes of animals treated with MP and/or Cd. Slides were counterstained with DAPI-fluorescent nuclear staining (blue). Scale bars represent 20 μm. Asterisk: LC. **(D)** Histogram showing the quantification of 3β-HSD fluorescence signal intensity, using ImageJ. All the values are expressed as means ± SEM from eight animals in each group. a vs. b *p* < 0.05; a vs. c *p* < 0.01; a vs. d *p* < 0.001; b vs. c *p* < 0.05; b vs. d *p* < 0.01; c vs. d *p* < 0.01. Each experiment has been performed in triplicate.

To assess the effects of MP and/or Cd on testicular steroidogenesis, serum T level, as well as 3β-HSD protein level and localization were evaluated (Figure 2). Data revealed that T concentration was significantly decreased in MP (*p* < 0.05), Cd + MP (*p* < 0.01) and Cd (*p* < 0.001) compared with controls (Figure 2A).

WB analysis performed on 3β-HSD, an enzyme involved in T biosynthesis, showed a decrease in its level in MP (*p* < 0.05), Cd + MP (*p* < 0.01) and Cd (*p* < 0.001) as compared to the controls (Figure 2B).

The impairment of T production was additionally confirmed by IF staining of 3β-HSD, shown in Figure 2C. For all groups analyzed, the signal specifically localized in the interstitial LC (asterisks, Figure 2C), but was fainter in the testis of treated animals. Fluorescence intensity analysis showed a similar, statistically significant trend to that observed for protein level (Figure 2D).

For all the considered parameters, the values were lower in the Cd group.

### 3.3 Effect of MP and/or Cd on apoptosis

**FIGURE 3:**
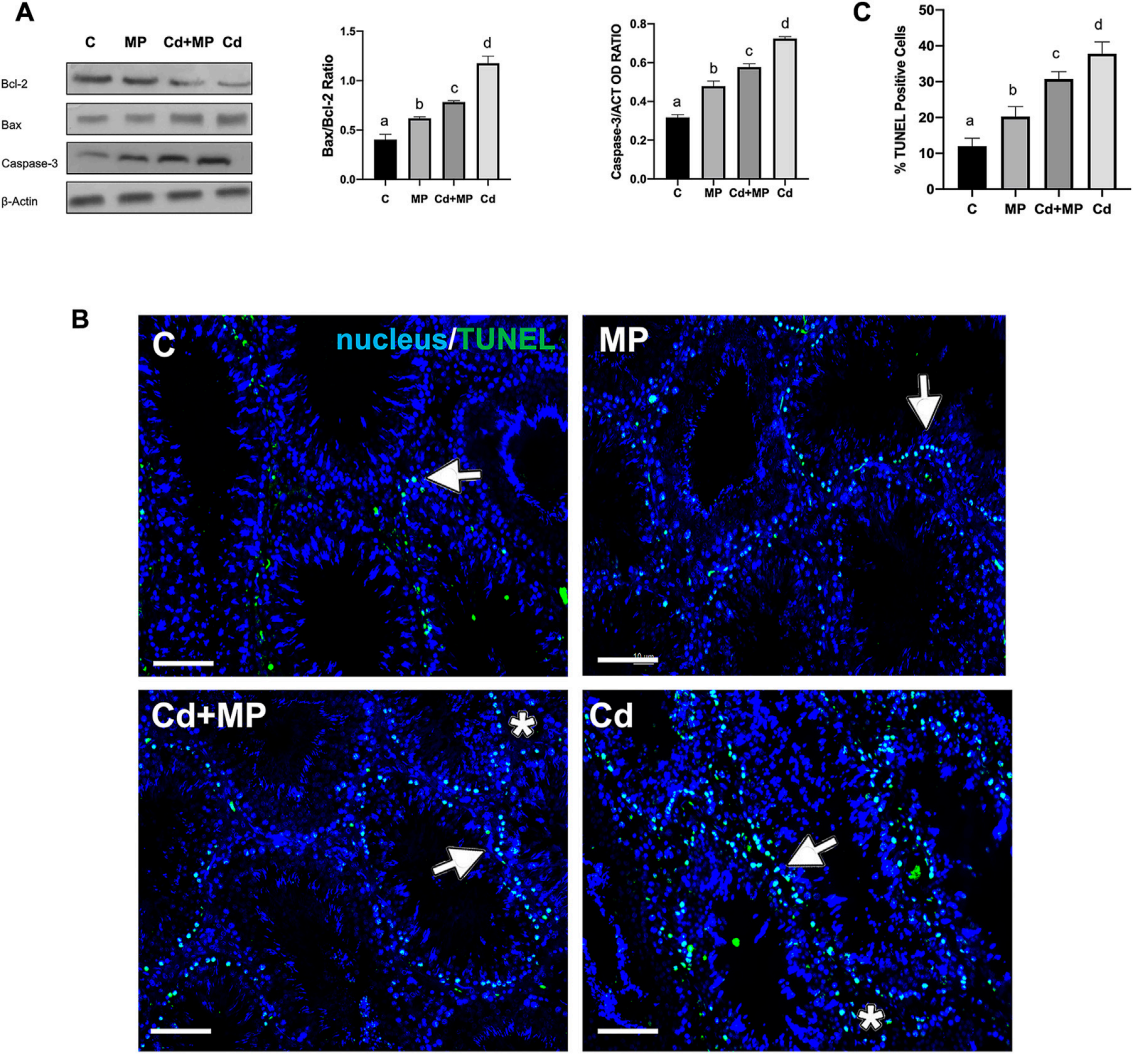
Apoptosis rate analysis of control, MP and/or Cd-treated rat testis. **(A)** WB analysis showing the expression of Bcl-2 (26 kDa); Bax (23 kDa) and Caspase-3 (31 kDa) in the testes of animals treated with MP and/or Cd. Histograms show the Bax/Bcl-2 ratio and Caspase-3 relative protein levels, respectively. Data were normalized with *ß*-Actin and reported as OD ratio. **(B)** Determination of apoptotic cells through the detection of TUNEL-positive cells (green) in the testes of animals treated with MP and/or Cd. Slides were counterstained with DAPI-fluorescent nuclear staining (blue). Scale bars represent 20 μm. Arrows: SPG; Asterisks: LC. **(C)** Histogram showing the % of TUNEL positive cells. All the values are expressed as means ± SEM from eight animals in each group. a vs. b *p* < 0.05; a vs. c *p* < 0.01; a vs. d *p* < 0.001; b vs. c *p* < 0.05; b vs. d *p* < 0.01; c vs. d *p* < 0.01. Each experiment has been performed in triplicate.

Figure 3 shows the effect of MP administration, alone or together with Cd, on the apoptotic rate of germ and somatic cells. WB analysis revealed an increase in Bax/Bcl-2 ratio and Caspase-3 protein level in the MP (*p* < 0.05), Cd + MP (*p* < 0.01) and Cd (*p* < 0.001) groups as compared to the control (Figure 3A).

In support of these data, a TUNEL assay was performed on all samples (Figure 2B). Data showed dispersed apoptotic cells in the control group, mainly SPG (arrows), and scattered LC (asterisks). As expected, in MP (*p* < 0.05) Cd + MP (*p* < 0.01) and Cd (*p* < 0.001) groups, the number of apoptotic cells increased dramatically, as compared to the control (Figures 2B,C). However, Cd treatment provoked more apoptosis than MP (*p* < 0.05) and Cd + MP (*p* < 0.01).

### 3.4 Effect of MPs and/or Cd on PTMA

**FIGURE 4:**
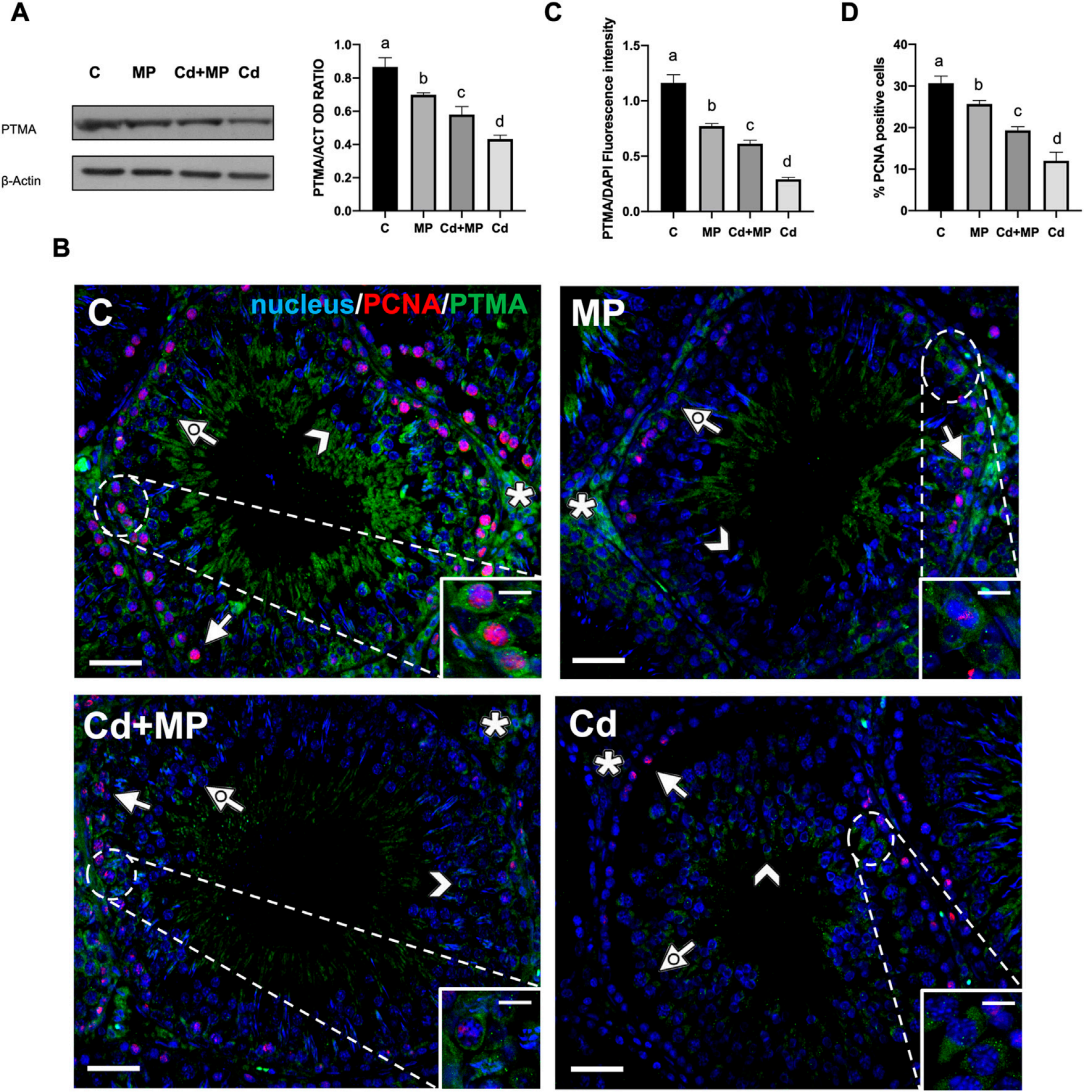
WB and IF analysis of PTMA and PCNA of control, MP and/or Cd-treated rat testis. **(A)** WB analysis showing the expression of PTMA (15 kDa) in the testes of animals treated with MP and/or Cd. Histogram shows its relative protein level. Data were normalized with *ß*-Actin and reported as OD ratio. **(B)** IF analysis of PTMA (green) and PCNA (red) in the testes of animals treated with MP and/or Cd. Slides were counterstained with DAPI-fluorescent nuclear staining (blue). Scale bars represent 20 μmand 10 μm in the insets. Arrow: SPG; dotted arrow: SPC; arrowhead: SPT; asterisk: LC. **(C)** Histogram showing the fluorescence signal intensity of PTMA. Data were normalized with DAPI signal. **(D)** Histogram showing the % of PCNA positive cells. All the values are expressed as means ± SEM from eight animals in each group. a vs. b *p* < 0.05; a vs. c *p* < 0.01; a vs. d *p* < 0.001; b vs. c *p* < 0.05; b vs. d *p* < 0.01; c vs. d *p* < 0.01. Each experiment has been performed in triplicate.

WB analysis showed that PTMA protein level decreased in MP (*p* < 0.05), Cd + MP (*p* < 0.01) and Cd (*p* < 0.001) as compared to the controls (Figure 4A).

The observed decrease was further confirmed by an IF analysis of PTMA, performed together with PCNA, a commonly used proliferation marker, on all samples (Figure 4B). In the control group, PTMA mainly localized in the perinuclear cytoplasm in SPG (arrows and insets), SPC (dotted arrows) and SPT (arrowheads). In addition, a positive signal was also observed in the interstitial LC (asterisks). In all treated groups, PTMA maintained its localization, but the signal intensity decreased significantly in MP (*p* < 0.05), Cd + MP (*p* < 0.01) and Cd (*p* < 0.001) as compared to the controls (Figure 4C).

As for PCNA, it was localized in the nucleus of proliferating cells as the layer of SPG at basal tubules in all groups (Figure 4B). However, the number of PCNA-positive cells showed an opposite trend to that observed for thef TUNEL-positive cells described in the previous paragraph, as it was lower in MP (*p* < 0.05), Cd + MP (*p* < 0.01) and Cd (*p* < 0.001) than in controls (Figure 4D). Again, Cd had the worst effects, as compared to MP and Cd + MP.

### 3.5 Effect of MP and/or Cd on DAAM1

**FIGURE 5:**
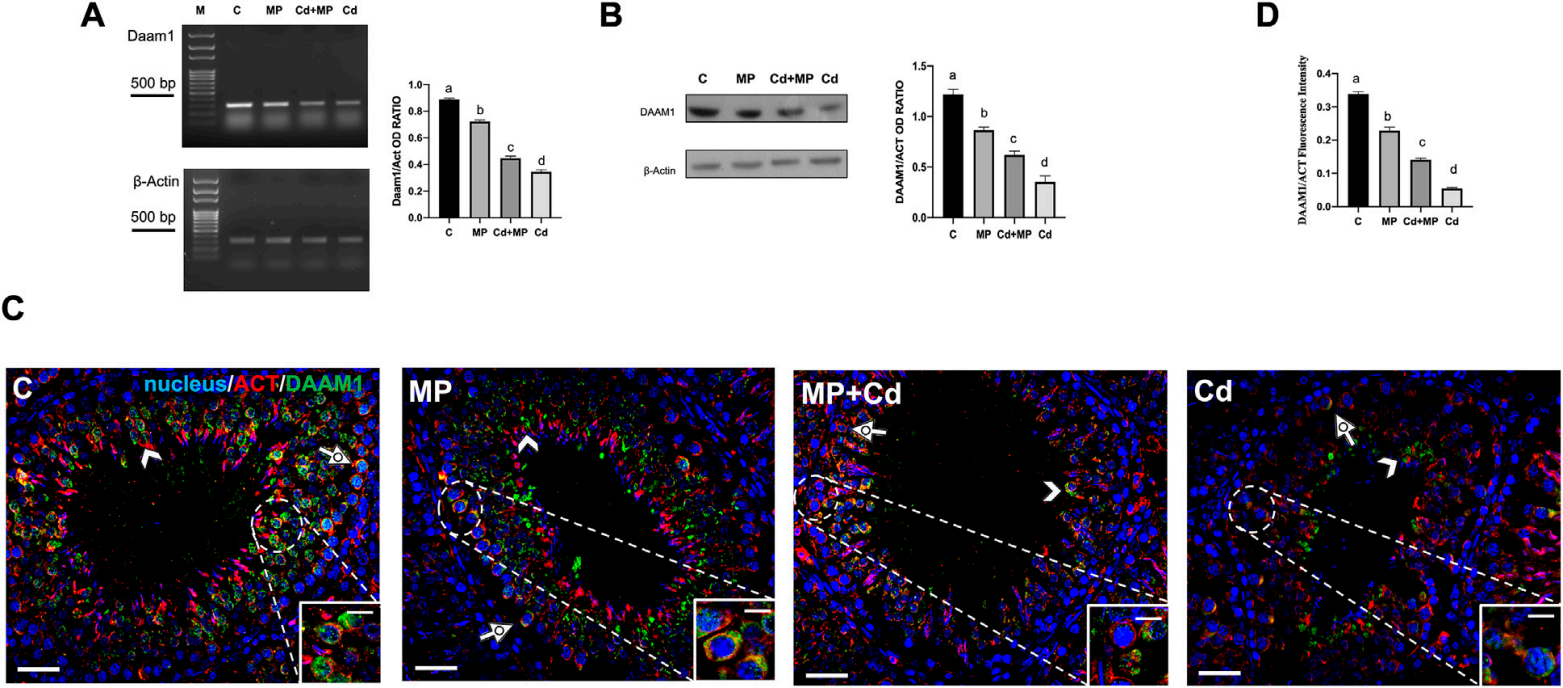
DAAM1 expression and localization in control, MP and/or Cd-treated rat testis. **(A)** Agarose gel electrophoresis of RT-PCR products showing the expression of Daam1 (380 bp), in testes of animals treated with MP and/or Cd. M: represents base-pairs marker (Solis BioDyne, Tartu, Estonia). Histogram shows the relative mRNA levels of Daam1. Data were normalized with ß-actin and reported as OD ratio. **(B)** WB analysis showing the protein levels of DAAM1 (112 kDa) in testes of animals treated with MP and/or Cd. Histogram shows its relative protein level. Data were normalized with ß-Actin and reported as OD ratio. **(C)** IF analysis of DAAM1 (green) and ß-Actin (red) in testes of animals treated with MP and/or Cd. Slides were counterstained with DAPI-fluorescent nuclear staining (blue). Scale bars represent 20 μm and 10 μm in the insets. Dotted arrows: SPC; arrowheads: SPT. **(D)** Histogram showing the quantification of DAAM1 fluorescence signal intensity; data were normalized with ß-Actin signal. All the values are expressed as means ± SEM from eight animals in each group. a vs. b *p* < 0.05; a vs. c *p* < 0.01; a vs. d *p* < 0.001; b vs. c *p* < 0.05; b vs. d *p* < 0.01; c vs. d *p* < 0.01. Each experiment has been performed in triplicate.

RT-PCR and WB analyses were performed on DAAM1 (Figures 5A,B). Although it is less sensitive than real-time qPCR, semiquantitative RT-PCR is a specific technique that allows us to appreciate changes in the expression level of target genes.

Analysis of Daam1 mRNA levels showed that its expression was downregulated by treatments. Specifically, the level of Daam1 decreased significantly in MP (*p* < 0.05), Cd + MP (*p* < 0.01) and Cd (*p* < 0.001) as compared to the controls (Figure 5A). WB analysis revealed a similar trend (Figure 5B). In fact, exposure to MP (*p* < 0.05), Cd + MP (*p* < 0.01) and Cd (*p* < 0.001) reduced the level of DAAM1 protein as compared to the control, similar to what was described previously.

To localize DAAM1 in the gonads of rats treated with MP and/or Cd, IF double staining was made along with its cytoskeletal partner, actin, (Figure 5C). In sections of control testis, DAAM1 localized in the perinuclear cytoplasm of SPC (dotted arrows, inset), and SPT (arrowheads), colocalizing with ß-actin, as evidenced by the yellow-orange intermediate staining. In the GC of treated animals, a drastic decrease of DAAM1 staining intensity was observed, with red signal predominating, however, the formin and *ß*-actin continued to colocalize in the perinuclear zone of SPC (dotted arrows). Fluorescence intensity analysis showed a comparable statistically significant trend, as previously specified for the protein level (Figure 5D).

Interestingly, also in this case, the effects of Cd on DAAM1 were the worst induced.

### 3.6 Effect of MP and/or Cd on PREP

**FIGURE 6:**
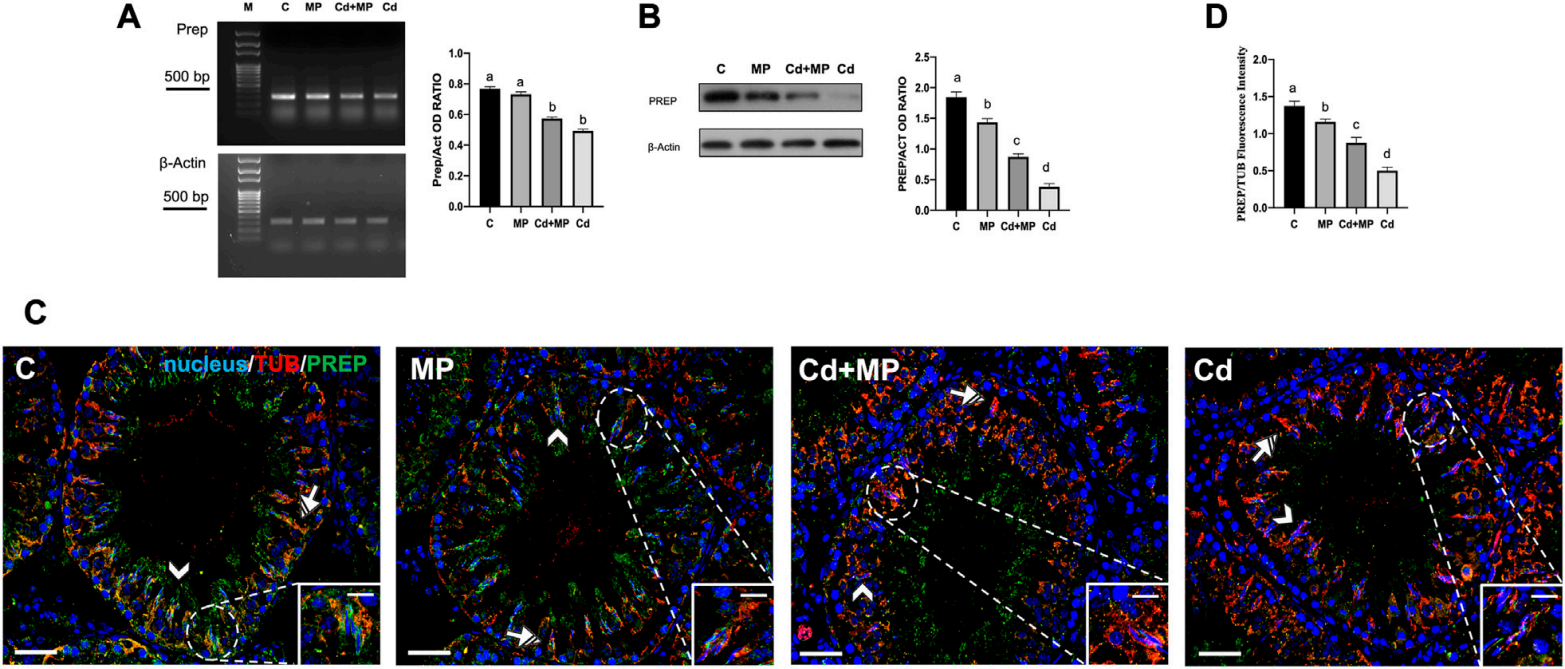
PREP expression and localization in control, MP and/or Cd-treated rat testis. **(A)** Agarose gel electrophoresis of RT-PCR products showing the expression of PREP (392 bp), in testes of animals treated with MP and/or Cd. M: represents base-pairs marker (Solis BioDyne, Tartu, Estonia). Histogram shows the relative mRNA levels of Prep. Data were normalized with β-actin and reported as OD ratio. **(B)** WB analysis showing the protein levels of PREP (80 kDa) in testes of animals treated with MP and/or Cd. Histogram shows its relative protein level. Data were normalized with β-Actin and reported as OD ratio. **(C)** IF analysis of PREP (green) and α-Tubulin (red) in testes of animals treated with MP and/or Cd. Slides were counterstained with DAPI-fluorescent nuclear staining (blue). Scale bars represent 20 μm and 10 μm in the insets. Arrowheads: SPT; striped arrows: SC. **(D)** Histogram showing the quantification of PREP fluorescence signal intensity, data were normalized with α-Tubulin signal. All thje values are expressed as means ± SEM from eight animals in each group. a vs. b *p* < 0.05; a vs. c *p* < 0.01; a vs. c *p* < 0.001; b vs. c *p* < 0.05; b vs. d *p* < 0.01; c vs. d *p* < 0.01. Each experiment has been performed in triplicate.

Figure 6 shows the effects of MP and or Cd treatments on PREP expression and localization.

In contrast to what was observed for Daam1, the mRNA level of Prep was downregulated only by Cd treatment, as no differences were observed between the MP and control, and Cd + MP and Cd groups (Figure 6A). PREP protein level decreased in all experimental groups, as the values in the MP (*p* < 0.05), Cd + MP (*p* < 0.01) and Cd (*p* < 0.001) groups were lower than those in the control (Figure 6B).

Immunolocalization of PREP showed that in the control it localized predominantly within the SPT (arrowheads) and the cytoplasmic extensions of SC, where colocalization with tubulin was evidenced by the yellow-orange intermediate color (striped arrows; insets). In sections from animals exposed to MP and or Cd, the distribution pattern of PREP was equivalento to that of the control and, but a decrease in staining was evident in GC (arrowheads) and in the SC cytoplasm (striped arrows). Finally, the analysis of the fluorescence intensity exhibited a similar, statistically significant trend to that above described.

From the analysis of the all the data, it is evident that the treatment with Cd alone produced the worst effects on PREP.

## 4 Discussion

In this paper we evaluated, for the first time, the combined effect of MP and Cd on the rat testis. We used 60-days-old rats, that, at this time-point, cannot be defined adult yet but in period of the late puberty/early adulthood. However, it has been well recognized that rats are sexually mature at this stage, as the first wave of the spermatogenic cycle is completed, and they can produce mature SPZ. In fact, the two signs of sexual maturity are the presence of complete spermiogenesis in the seminiferous tubules and mature SPZ in the epididymis (Picut et al., 2018).

Currently, especially in the most industrialized states, there is a critical impairment in the quantity and quality of SPZ, implying that the discharge of many pollutants into the environment is a major contributor to reduced fertility (Selvaraju et al., 2021). Furthermore, according to WHO guidelines (WHO, 2021), the seminogram, the analysis of macroscopic (volume, pH, viscosity) and microscopic (concentration, morphology, vitality, and motility of SPZ) characteristics is, to date, the best and most routinely used test for the assessment of male fertility; on the other hand, this test is not the most accurate, because seminal parameters within the proposed reference range do not promise fertility, nor do values outside these limits automatically indicate male infertility (Serrano et al., 2022). Thus, more detailed analyses of the molecular mechanism(s) of testis and SPZ physiology are mandatory not only to expand knowledge of spermatogenesis but also, by identifying new molecular markers, to improve the standard assessment of male fertility through routine semen analysis. Since many pollutants exert their harmful effects through proteins and signaling pathways that are also involved in the regulation of the testicular activity, the use of *in vivo* and *in vitro* treated-models may be useful not only to manage reproductive dysfunction, but also to better elucidate the mechanisms that support spermatogenesis under physiological conditions (Gao et al., 2021).

In this paper, the combined, injurious effect of MP and Cd on the rat testis was evaluated.

It is well known that, in the Wistar rats’ spermatogenesis lasts about 63 days, including SPZ maturation during epididymal transit (Picut et al., 2018). Although to observe a more accurate impact of the used pollutants on sperm quality, the treatment should have lasted at least 63 days, in this paper, in any case, alterations were observed in all the cell types that compose the SE, and in the mature SPZ. Thus, it can be assumed that all cells, even those that have already started mitotic and meiotic divisions, as well as spermiogenesis, are susceptible to MP and/or Cd effects, independently of the duration of treatment.

Here, in support of data shown in other papers, we confirm that both MP (Jin et al., 2022; Wei et al., 2022; Wen et al., 2022) and Cd (Zhu et al., 2020; Venditti et al., 2021c; Kechiche et al., 2021; Ali et al., 2022) can act as EDCs. Indeed, the presence of scattered apoptotic LC and altered steroidogenesis, evidenced by the decreased serum T concentration and 3β-HSD protein level, result in impaired central and local steroid availability and production. It should be noted that T, acting as a survival factor, controls the entire spermatogenetic process, preventing GC entry into apoptosis, therefore, its reduction may be an additional cause of induced apoptosis (Huhtaniemi, 2018; Venditti et al., 2021d). This last point was also confirmed by the increase in the Bax/Bcl-2 ratio and caspase-3 levels, as well as the decrease in the number of PCNA positive cells, indexes of the pro-apoptotic state and reduced proliferation, respectively.

In addition, our results showed, for the first time, that PTMA is a target protein of the harmful effects of MP and Cd. PTMA is a peculiar small polypeptide that, due to its unfolded structure, can interact with several molecules, participating in different biological pathways and activities, such as cell cycle regulation (Karachaliou et al., 2020). Indeed, PTMA has been demonstrated to act as an anti-apoptotic factor, inhibiting apoptosome formation (Zhang et al., 2014), or, through its association with the c-myc oncogene, promoting cell cycle progression (Boán et al., 2001). Therefore, the increase in apoptosis observed following MP and Cd treatments could also be related to the reduction in PTMA levels. It should also be mentioned, regarding spermatogenesis, that previous works have reported the specific association of PTMA with male gametes differentiation and, in particular, with acrosome biogenesis and early fertilization events (Ferrara et al., 2010; 2013; Pariante et al., 2016b; Venditti and Minucci, 2017). Thus, MP- and/or Cd-induced reduction of its protein levels could consequently result in impaired gamete quality and function.

One of the most important features of the spermatogenic process is its dynamics, characterized by proliferative and differentiative phases, during which round SPG intimately change their morphology and shape to produce mature SPZ. This is possible mainly due to the cytoskeleton of somatic and germ cells, as well as the hundreds of associated proteins that regulate the appropriate organization and activity of cytoskeletal elements (Li et al., 2017).

First, we confirmed that Cd can have a negative impact on the activity of many transcription factors (Venditti et al., 2021b), as the downregulation of DAAM1 and PREP expression was also observed here. Second, the results obtained showed that MP can also have an influence on DAAM1 and PREP; however, whereas for DAAM1 MP alone were able to decrease its mRNA level, for PREP such decrease was observed only for the protein. Therefore, given the role of these two proteins in the cytoskeletal remodeling that occurs during GC differentiation, we hypothesize that the observed impaired histological features, such as loss of contact and the presence of abundant empty spaces between cells, as well as the damaged morphometric parameters, may be due to a reduced expression of DAAM1 and PREP induced by MP and Cd treatment.

Interestingly, regarding PREP, in our previous work we found an increase of its expression induced by Cd (Venditti et al., 2020d). This could be justified with differences in the experimental design and in the age of the rats. Indeed, previously, rats were not directly exposed to Cd, but their mother were, during gestation and lactation (Venditti et al., 2020d), whereas, here, adult male rats were treated. Considering that effects of Cd on the function of the hypothalamus-pituitary-testis axis is age-dependent (Lafuente et al., 2000; 2001), and that PREP expression and activity changes in the rat brain during embryonal and post-natal development (Agirregoitia et al., 2010; Hannula et al., 2011), the divergent results may be attributed to the described varied Cd action and the changes of PREP levels during rats’ development.

Finally, the combined effects of Cd + MP were more intense than those induced by MP, but better than those induced by Cd alone. This antagonistic effect has been previously demonstrated in many experimental models and conditions, as MP reduced Cd toxicity in plants (Lian et al., 2020; Wang et al., 2022b; Zhang et al., 2022), fish (Wang S. et al., 2022; Yang et al., 2022), and mice (Wang et al., 2022d). Considering that the two substances were administered separately (oral gavage for MP and drinking water for Cd) and that the rates of adsorption/release of heavy metals by MP depend on pH values (Hildebrandt et al., 2021; Chen et al., 2022), we hypothesized that MP, by exploiting a sort of “positive-Trojan horse effect”, may reduce the bioavailability of Cd, explaining the minor consequences observed by concomitant treatment with MP-Cd as compared with Cd alone. Therefore, current studies focus on the use of “pre-adsorbed” MP with Cd to evaluate an effective “Trojan horse” effect on the rat testis.

## 5 Conclusion

In this paper, we further documented the combined effects of MP and Cd on rat testis physiology. Particular attention was given to their impact on SE histology, hormonal milieu, apoptosis, and, for the first time, on the expression of the two cytoskeleton-associated proteins DAAM1 and PREP. Our data confirm that cytoskeleton elements are a major target of environmental pollutants, resulting, ultimately, in impaired testicular activity.

Interestingly, because MP and Cd were administered to rats contemporaneously, but through two separate ways, the observed alterations were less than for Cd alone, probably due to reduced bioavailability of this heavy metal. Finally, this work emphasizes the role of proteins that regulate cytoskeletal dynamics in the proper progression of spermatogenesis.

## Data Availability

The raw data supporting the conclusion of this article will be made available by the authors, without undue reservation.

## Abbreviations

3β-HSD, 3β-Hydroxysteroid dehydrogenase; Bax, Bcl-2 Associated X; Bcl-2, B-cell lymphoma 2; BSA, bovine serum albumin; BTB, blood-testis barrier; Cd, cadmium; DAAM1, dishevelled-associated activator of morphogenesis 1; DAPI, 4′,6-diamidino-2-phenylindole; mEDCs, endocrine disrupting chemicals; GC, germ cells; IF, immunofluorescence; LC, Leydig cells; MP, microplastics; OD, optical density; PCNA, proliferating cell nuclear antigen; PREP, prolyl endopeptidase; PTMA, prothymosin α; SC, Sertoli cells; SE, seminiferous epithelium; SPG, spermatogonia; SPC, spermatocytes; SPT, spermatids; SPZ, spermatozoa; T, testosterone; TUNEL, Terminal Transferase dUTP Nick End Labeling; WB, Western blotting.
