# Supplementary material for: The simultaneous administration of microplastics and cadmium alters rat testicular activity and changes the expression of PTMA, DAAM1 and PREP
Source: Front Cell Dev Biol. 2023 Mar 9;11:1145702. doi: 10.3389/fcell.2023.1145702 (PMC10033688; doi:10.3389/fcell.2023.1145702)
Supplement: Supplementary file 1 [file Table1.DOCX]

**Table S1.** List of all the used primers.

| **Gene** | **GenBank**  **Accession Nr.** | **Annealing Temperature** | **Amplicon size (bp)** | **Primers Sequence** |  |
| --- | --- | --- | --- | --- | --- |
| Daam1 | NM_001108030.1 | 56° C | 380 | For: 5’- CCGAAACAATGACCACCCAG -3’  Rev: 5’- GCTTTGTCCTCAGTGCTGTC -3’ |  |
| Prep | NM_031324.2 | 55° C | 392 | For: 5’‐ CCCTTATGCTTGGCTTGAAG ‐3’  Rev: 5’‐ TCATGAACTTGATGGTCACC ‐3’ |  |
| β-Actin | NM_031144.3 | 56° C | 300 | For: 5’- CTCTTCCAGCCTTCCTTCCT -3’  Rev: 5’- CTGCTTGCTGATC-CACATC -3’ |  |
