# Supplementary material for: The simultaneous administration of microplastics and cadmium alters rat testicular activity and changes the expression of PTMA, DAAM1 and PREP
Source: Front Cell Dev Biol. 2023 Mar 9;11:1145702. doi: 10.3389/fcell.2023.1145702 (PMC10033688; doi:10.3389/fcell.2023.1145702)
Supplement: Supplementary file 2 [file Table2.DOCX]

**Table S2.** List of all the used antibodies.

| **Antibody** | **WB Dilution** | | **IF Dilution** | **Source** |  |
| --- | --- | --- | --- | --- | --- |
| 3β-HSD | 1:700 | | 1:100 | Elabscience Biotechnology, Wuhan, China  #E-AB-15112 |  |
| Bcl-2 | 1:500 | | - | Elabscience Biotechnology, Wuhan, China  #E-AB-60012 |  |
| Bax | 1:1000 | | - | Elabscience Biotechnology, Wuhan, China  #E-AB-13814 |  |
| Caspase-3 | 1:700 | | - | Elabscience Biotechnology, Wuhan, China  #E-AB-22115 |  |
| PTMA | 1:500 | | 1:100 | Abcam, Cambridge, UK  #ab247074; |  |
| PCNA | - | | 1:100 | Thermo Fisher Scientific, Waltham, MA, USA  #MA5-11358 |  |
| DAAM1 | 1:1000 | | 1:100 | Elabscience Biotechnology, Wuhan, China  #E-AB-21207 |  |
| PREP | 1:3000 | | 1:100 | Abcam, Cambridge, UK  #ab58988 |  |
| β-Actin | 1:5000 | | 1:100 | Elabscience Biotechnology, Wuhan, China  #E-AB-20031 |  |
| α-Tubulin | - | | 1:100 | Elabscience Biotechnology, Wuhan, China  #E-AB-20036 |  |
| Goat anti-rabbit HRP | 1:5000 | | - | Sigma-Aldrich, Milan, Italy  #AP307P |  |
| Goat anti-mouse HRP | 1:10000 |  | - | Sigma-Aldrich, Milan, Italy  #AP130P | |
| Goat anti-rabbit  Alexa Fluor 488 | - |  | 1:500 | Thermo Fisher Scientific, Waltham, Ma, USA  #A32731 | |
| Goat anti-mouse  Alexa Fluor 647 | - |  | 1:500 | Thermo Fisher Scientific, Waltham, Ma, USA  #A21236 | |
